# Patient-centered placement matching of alcohol-dependent patients based on a standardized intake assessment: process evaluation within an exploratory randomized controlled trial

**DOI:** 10.1186/s12888-022-03705-9

**Published:** 2022-01-27

**Authors:** Angela Buchholz, Michael Berner, Judith Dams, Anke Rosahl, Jochen Hempleman, Hans-Helmut König, Alexander Konnopka, Levente Kriston, Daniela Piontek, Jens Reimer, Jeanette Röhrig, Norbert Scherbaum, Anna Silkens, Ludwig Kraus

**Affiliations:** 1 Department of Medical Psychology, Centre for Psychosocial Medicine, University Medical Centre of Hamburg-Eppendorf, 20246 Hamburg, Germany; 2 Municipal Clinical Center of Karlsruhe, Karlsruhe, Germany; 3 Department of Health Economics and Health Services Research, University Medical Centre Hamburg-Eppendorf, Hamburg, Germany; 4 Outpatient Department for Addiction, LWL-Hospital Muenster, Muenster, Germany; 5 IFT Institut für Therapieforschung, München, Germany; 6 Centre for Interdisciplinary Addiction Research, University of Hamburg, Hamburg, Germany; 7 Centre for Psychosocial Medicine, Health North, Bremen, Germany; 8 Clinic for Addiction Medicine and Addictive Behaviour, Institute for Clinical Psychology, Klinikum Stuttgart, Stuttgart, Germany; 9 LVR-Hospital Essen, Department of Addictive Behavior and Addiction Medicine, Medical Faculty, University of Duisburg-Essen, Duisburg, Germany; 10 Department for Public Health Sciences, Stockholm University, Stockholm, Sweden; 11 Institute of Psychology, ELTE Eötvös Loránd University, Budapest, Hungary

**Keywords:** Process evaluation, Patient-centred placement matching, Allocation guidelines, Measurement in the Addictions for Triage and Evaluation, Comprehensive assessment, Health-services research

## Abstract

**Background:**

In the implementation of placement matching guidelines, feasibility has been concerned in previous research. Objectives of this process evaluation were to investigate whether the patient-centered matching guidelines (PCPM) are consistently applied in referral decision-making from an inpatient qualified withdrawal program to a level of care in aftercare, which factors affect whether patients actually receive *matched* aftercare according to PCPM, and whether its use is feasible and accepted by clinic staff.

**Methods:**

The study was conducted as process evaluation within an exploratory randomized controlled trial in four German psychiatric clinics offering a 7-to-21 day qualified withdrawal program for patients suffering from alcohol dependence, and with measurements taken during detoxification treatment and six months after the initial assessment. PCPM were used with patients in the intervention group by feeding back to them a recommendation for a level of care in aftercare that had been calculated from Measurements in the Addictions for Triage and Evaluation (MATE) and discussed with the staff on the treatment unit. As measurements, The MATE, the Client Socio-Demographic and Service Receipt Inventory—European Version, a documentation form, the Control Preference Scale, and the Motivation for Treatment Scale were administered. A workshop for the staff at the participating trial sites was conducted after data collection was finished.

**Results:**

Among 250 patients participating in the study, 165 were interviewed at follow-up, and 125 had received aftercare. Although consistency in the application of PCPM was moderate to substantial within the qualified withdrawal program (Cohen’s kappa ≥ .41), it was fair from discharge to follow-up. In multifactorial multinomial regression, the number of foregoing substance abuse treatments predicted whether patients received more likely undermatched (Odds Ratio=1.27; *p*=.018) or overmatched (Odds Ratio=0.78; *p*=.054) treatment. While the implementation process during the study was evaluated critically by the staff, they stated a potential of quality assurance, more transparency and patient-centeredness in the use of PCPM.

**Conclusions:**

While the use of PCPM has the potential to enhance the quality of referral decision making within treatment, it may not be sufficient to determine referral decisions for aftercare.

**Trial Registration:**

German Clinical Trials Register DRKS00005035. Registered 03/06/2013.

## Introduction

Alcohol use disorders and its consequences include a wide range of potential limitations in medical and psychosocial functioning [1, 2] and need tailored and flexible treatment strategies. Therefore, placement matching seems a promising approach in substance abuse treatment choosing for each patient an optimal treatment intensity (level of care), based on a comprehensive assessment of the patients’ needs and the best available evidence while respecting the patients’ preferences. One important hypothesis of placement matching approaches is that treatment outcome should improve when patients are matched to an appropriate level of care compared to a less intensive one (*undermatched*). Conversely, there should be no added value if patients are *overmatched* and receive more treatment than appropriate (e.g. [3, 4]). In order to provide feasible and valid support for clinical decision making, different placement matching guidelines have been developed for substance abuse treatment. Usually, they comprise a set of criteria and decision rules resulting in a treatment recommendation for each patient by means of an algorithm. To gather all relevant clinical information for using placement matching guidelines, standardized assessments are being recommended, frequently aided by computer-assisted tools [5, 6].

However, placement matching approaches have been supported in the literature (e.g. [7, 8]). There is evidence for matching treatment to the patients’ needs [9, 10] and to treatment intensities or different settings [11, 12], but the underlying hypotheses of matching have not always been fully supported [3, 4]. Furthermore, potential risks or side effects of under- or overmatching have not been investigated so far. In general, side effects of psychotherapy are not sufficiently investigated [13].

As a European alternative to the Patient Placement Criteria developed by the American Society of Addiction Medicine [14], Dutch matching guidelines have been developed and evaluated [15, 16], including the Measurements in the Addictions for Triage and Evaluation (MATE) as assessment interview [17]. In close cooperation with the Dutch study group, we translated the MATE into German language [18, 19] and proposed adapted placement matching guidelines called *patient-centered placement matching guidelines* (PCPM) that can be used to facilitate treatment decision making in the German substance abuse treatment [20, 21]. PCPM include three consecutive stages [20]: In stage A (*treatment entry*), a clinician reviews the patients’ current desire for help and his or her preferences for treatment. Furthermore, indication criteria for the use of the PCPM are evaluated. If the patient wishes a referral to SAT and there are no contraindications, in stage B (*recommendation to a LOC based on MATE dimension scores*), a MATE interview is conducted. Based on the four *MATE dimension scores*, one of four levels of care (LOC; LOC1: brief outpatient advice; LOC2: outpatient treatment; LOC3: day/residential treatment; LOC4: in- or outpatient long-term care) can be recommended. During stage C *(allocation talk)*, the recommendation is fed back to the patient and discussed considering all factors that may have an effect on the treatment decision, e.g. motivation or preferences of the patient or organizational factors [20]. While previous studies regarding placement matching have been conducted retrospectively or have used naturalistic designs [3], we used a rigorous randomized controlled design to investigate whether the use of PCPM in an inpatient qualified withdrawal treatment [22] is more effective in reducing heavy drinking and costs 6 months after discharge from an inpatient alcohol withdrawal treatment compared to usual referral to aftercare [23, 24].

However, some previous findings suggested a lack of feasibility in the implementation of placement matching guidelines into routine care [5, 6, 15]. With regard to the technical conditions, obstacles can occur due to the structure and quality of data or a lack of compatibility with existing electronic patient record systems [25]. Given the high complexity of implementing placement matching guidelines in routine decision-making procedures and its sensitivity to regional variations, it can be regarded as complex intervention [26]. That means, process evaluation research can be highly valuable in order to identify potential causal chains that link the use of PCPM with health outcomes and costs. Our randomized controlled trial was designed with a strong emphasis on process research. While we did not find main effects of PCPM use on both primary outcomes heavy drinking and costs, we found support for our hypothesized effect mechanism: Patients who received *matched* aftercare reported significantly fewer days of heavy drinking than *undermatched* patients. For patients who were *overmatched*, direct costs were significantly higher with no additional effects on alcohol consumption compared to *matched* patients [23]. Since matched patients were equally distributed between intervention group and control group, PCPM may not have been successfully implemented in the intervention group. Results of our process evaluation may help to get a better understanding of the underlying effect mechanisms. Our process evaluation had three major objectives:

(1)Assessing consistency of PCPM: A necessary precondition to a potential effect of the PCPM on matching and health outcomes is that recommendations regarding a LOC remain consistent throughout the different stages of PCPM.

(2)Investigating factors affecting matching: Several factors may affect whether patients receive matched, overmatched or undermatched aftercare, i.e., organizational and reactive factors. Information regarding these factors may be useful to derive recommendations for an effective implementation of PCPM.

(3)Gathering information regarding feasibility and acceptance of PCPM from the view of the staff: Information regarding acceptance and feasibility is necessary in order to get a better understanding of the study results on the one hand and is useful in order to suggest ways to implement PCPM in routine care on the other hand.

## Methods

### Study design and setting

We used a parallel two-arm randomized controlled trial design with assessments in the first week of treatment and six months after the initial assessment interview. In the intervention group, an additional assessment was performed immediately after the intervention. Besides the telephone-based follow-up interview, all assessments were conducted during the qualified withdrawal treatment. Participating study sites were located at four psychiatric clinics in different regions of Germany offering inpatient alcohol withdrawal treatment in a specialized treatment unit. Inclusion criteria for patients were being admitted to alcohol withdrawal treatment and having a primary diagnosis of alcohol dependency. Exclusion criteria comprised being in treatment for reasons other than alcohol dependence, in crisis and needing crisis intervention, severely cognitively impaired, psychotic, illiterate, or having insufficient German language skills. Patients were also excluded, when aftercare was already organized at start of the qualified withdrawal program. Due to the high patient turnover at the participating treatment units, a consecutive eligibility screening of patients could not be realized. Therefore, staff of the clinics pre-selected patients for eligibility screening by both practical considerations and in-and exclusion criteria of the study [23]. The trial was registered in the German Clinical Trials Register DRKS00005035 (03/06/2013).

### Implementation of PCPM

Since the PCPM makes no statement regarding its use in multidisciplinary teams, few aspects had to be specified for its use in our study. Within an inpatient treatment unit, recommendations and decisions regarding treatment and referral are usually discussed in a multidisciplinary team. In the use of PCPM, this mainly affects stage C *allocation talk* which was therefore subdivided into three different components: (1) discussion of the results with the multidisciplinary team, (2) feedback session with patient, and (3) reporting results back to the team. That means, a recommendation regarding referral to a LOC for aftercare was calculated using the MATE and reviewed with the team afterwards. This recommendation was discussed with the patient and a research assistant in a feedback session, and both agreed upon the appropriate LOC. Results of this feedback session were reported to the multidisciplinary team afterwards and patients of the intervention group continued in their qualified withdrawal program as usual. A closer description of the intervention can be found elsewhere [23].

### Process evaluation measures

Measures and data sources, that were used at different stages during the course of the trial used to gain process evaluation data are reported in the following. A complete description of all measures used in the study is published elsewhere [24].

(1)Treatment entry. Patients who participated in the study received a questionnaire including the Control Preference Scale, which is a single item measure to account for the patients’ preferred role in medical decision-making: active, passive or shared [27].

(2)Assessment interview. When withdrawal symptoms had been declined, i.e. usually 3-5 days after treatment entry, the MATE and the Client Sociodemographic and Service Receipt Inventory [28] (CSSRI-EU) were conducted in one interview session. The CSSRI-EU was used to assess data on health services utilization. Furthermore, patients completed the Motivation for Treatment Scale (MFT) [29]. The MFT is a 24-item questionnaire including four scales *problem recognition specific*, *problem recognition general*, *desire for help* and *treatment readiness*. Patient characteristics were assessed using a short documentation form.

(3)Intervention. The intervention procedure was documented using a documentation sheet. All LOC recommendations that were given to the patient during the stages of PCPM were documented during the intervention: This included (a) the LOC that was recommended based on the MATE, (b) possibly dissenting recommendations after discussion with the staff, and reasons for the disagreement, (c) results from the feedback session with the patients. Again, deviations from the recommended LOC were documented if they occurred.

(4)Treatment discharge. On a separate documentation sheet, referral decisions that were made at discharge from the qualified withdrawal treatment were recorded for both the intervention and control group.

(5)Follow-Up. Six months after the initial interview, the MATE and the CSSRI-EU were administered by phone.

(6)Expert workshop with trial sites. After the data collection was finished, all research assistants and staff members of the participating treatment units were invited to take part in an expert workshop. In the workshop, preliminary results of the study were presented. Afterwards, the group was asked to discuss feasibility and the potential of implementation of the PCPM in the German substance abuse treatment according to the following broad questions:

- Was the assessment feasible in routine practice?
- Were the study and the study procedure feasible?
- Was the PCPM plausible and feasible in practice?
- What potentials and challenges are to expect, when this approach should be implemented in routine care?

### Data preparation and statistical analyses

Recommended LOC at the different points of measurement (MATE-Interview, discharge, follow-up) were analyzed descriptively for both intervention and control group. For the intervention group, the recommended LOC after the staff discussion and after the feedback session were analyzed additionally. In order to examine consistency throughout the different components of the intervention, Cohens’ kappa (κ) was calculated. A kappa ≤0 was considered as poor agreement, 0-0.20 as slight agreement, 0.21-0.40 as fair agreement, 0.41-0.60 as moderate agreement 0.61-0.80 as substantial agreement, and 0.81-1.00 as (almost) perfect agreement [30].

Whether patients received matched or mismatched treatment in the follow-up period, has been calculated based on the CSSRI-EU assessment at follow-up. To do so, substance abuse treatment utilization was categorized to one of the four LOC independently by two researchers. By comparing the LOC recommended by the MATE interview (control group) or the LOC resulting from the feedback session (intervention group) with the LOC patients actually had received at follow-up, a matching variable was calculated. Herein, *undermatched* patients had received less treatment than recommended, *matched* patients had received the recommended treatment, and *overmatched* patients received more treatment than recommended (for more details see [23]). Factors with a potential influence on the matching process that were not part of the intervention (contextual information, reactive and organizational factors) were investigated using bivariate and multifactorial multinomial regression analyses with *matching* as dependent variables and the CPS [27], the MFT [29], the categorical MATE dimension-score history of substance use disorder treatment, the dichotomous MATE-dimension scores severity of addiction, severity of psychiatric comorbidity, severity of social disintegration, age, gender, and trial site as independent variables. For these analyses, only patients with complete data sets at all points of measurement that underwent any kind of (substance abuse) aftercare during the follow-up period were included.

Feasibility and acceptance were discussed in the expert workshop after the data collection was finished. The expert workshop was audiotaped, transcribed and analyzed using qualitative content analysis [31].

## Results

### Sample

From 1927 patients that were treated in all four study sites in the study period, a total of 299 were invited to participate in the study by research assistants. Of those, 250 patients participated and were randomized either to the intervention group (*n*=123) or the control group (*n*=127). Mean age of the patients was 45.2 (SD=10.32) with an average duration of heavy alcohol use of 13.2 years (SD=10.08). In Table 1, descriptive characteristics of the contextual, reactive and organizational factors included in further analyses are presented. Further details of participant flow and dropout are published elsewhere [23].

**Table 1:** Descriptive characteristics of the study sample at baseline

	Total (***N*** = 250)		Treated at follow-up* (***N*** = 127)	
	n	%	n	%
**Gender**				
*Female*	86	34.4	50	39.4
*Male*	164	65.6	77	60.6
**Motivation for Treatment**^**A**^	***M***	***SD***	***M***	***SD***
*Problem recognition – General*	4.01	0.89	4.05	0.85
*Problem recognition – Specific*	3.00	0.97	3.06	0.95
*Desire for Help*	3.53	0.77	3.53	0.74
*Treatment Readiness*	3.99	0.75	3.96	0.80
**Preferred Role in Decision making**^**B**^	121	48.4	54	42.5
*Active*	96	38.4	57	44.9
*Shared*	25	10.0	14	11.0
*Passive*	8	3.2	2	1.6
**Severity of addiction**^**C**^				
*High*	147	58.8	75	59.1
*Low*	103	41.2	52	40.9
**Severity of psychiatric comorbidity**^**C**^				
*High*	125	50.0	63	49.6
*Low*	125	50.0	64	50.4
**Severity of social disintegration**^**C**^				
*High*	75	30.0	14	32.3
*Low*	175	70.0	86	67.7
**History of substance use disorder treatment**^**D**^				
*0 - 1*	126	50.4	60	47.2
*2*	26	10.4	16	12.6
*3 – 5*	44	17.6	28	22.0
*> 6*	54	21.6	23	18.1
**Trial Site**				
*A*	73	29.2	37	29.1
*B*	56	22.4	27	21.3
*C*	48	19.2	23	18.1
*D*	73	29.2	40	31.5

*Only patients who received treatment at follow-up were included in the regression analyses regarding factors affecting the matching process^A^ Results of the four subscales of the Motivation for Treatment Scale; ^B^ Results of the Control Preference Scale ^C^Dichotomous dimension scores calculated from the Measurements in the Addictions for Triage and Evaluation (MATE); ^D^ Categorical score calculated from the MATE

### Consistency of PCPM

**Fig. 1:**
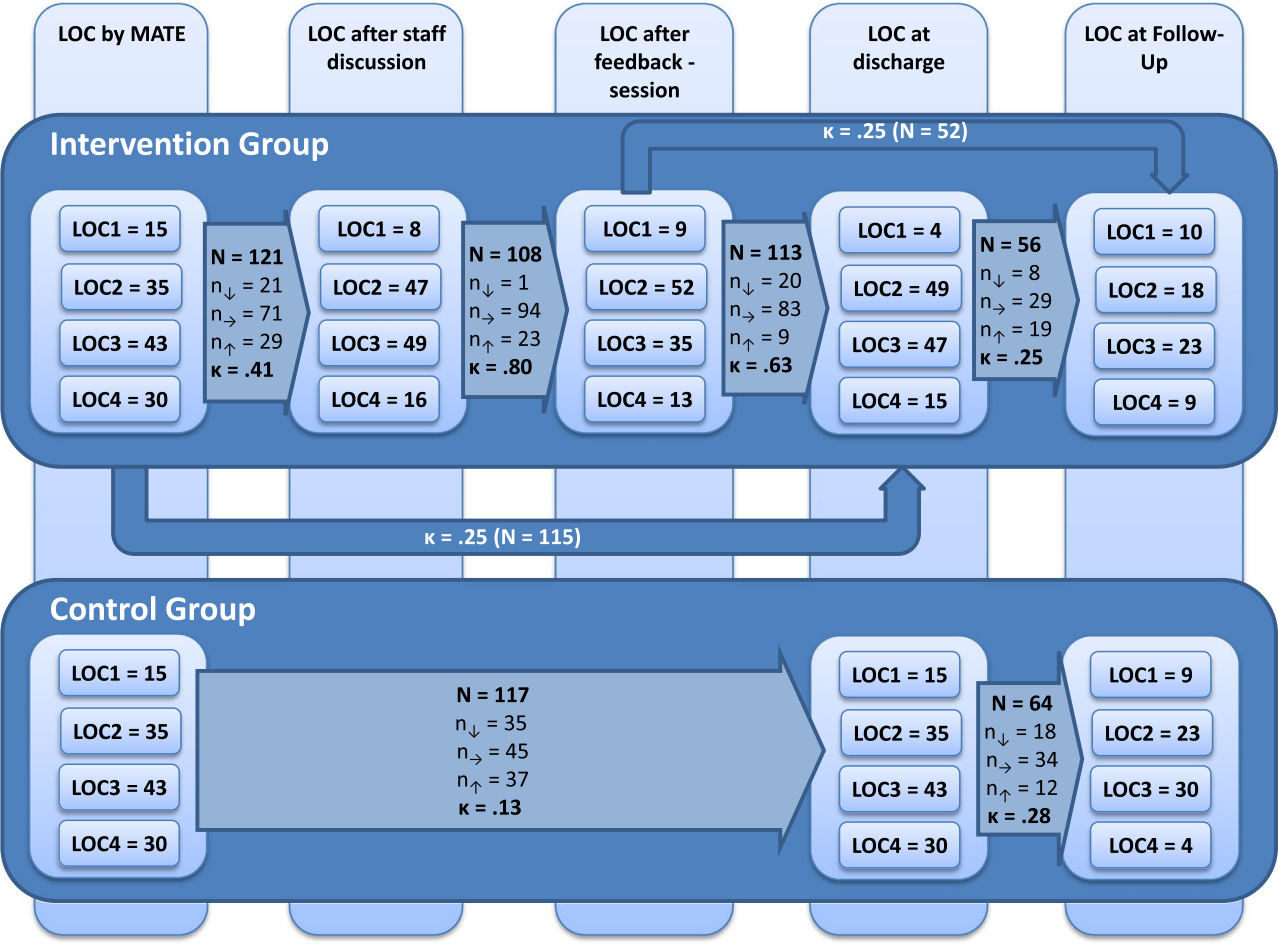
Consistency of recommendations for referral to aftercare throughout the qualified withdrawal treatment until follow-up assessment for the intervention and control group; LOC=Level of Care; K=Cohens Kappa; Consistencies that could be calculated for both groups IG and CG have also been published elsewhere [23]

Of the patients that were reached at follow-up, 19 (11.4%) received treatment according to LOC1, 41 (24.5%) according to LOC2, 53 (31.7%) according to LOC3, and 14 (8.4%) according to LOC4. About 40 (24.0) patients did not receive any substance abuse treatment, with more patients in the intervention group receiving no treatment compared to the control group (nIG =24; nCG=14; χ2=3.84; df=1; *p*=.050). In Fig. 1, all recommendations to LOCs at different points of measurement throughout the study are described for both, IG and CG. Compared to LOC1 and LOC4, LOC2 and LOC3 were recommended more frequently. There were no major differences between intervention and control group regarding the recommendations that were assessed for both groups (MATE-interview, discharge, and follow-up). In both, intervention and control group, agreement between LOC at discharge and follow-up was fair (κIG=.25, κCG=.28) whereas agreement between LOC at MATE-interview and discharge was fair in the intervention group (κIG=.25) and poor in the control group (κCG=.13) (see also 14).

In the intervention group, agreement was additionally calculated throughout all steps of the PCPM process. We found moderate agreement between the LOC-recommendation resulting from the MATE-interview and the LOC-recommendation after staff discussion (κ=.41). In 29 patients (24%), the staff changed the recommendation to a lower LOC than recommended by the MATE; in 21 patients (17%), the recommendation was altered to a higher LOC than recommended. Disagreement with the MATE-recommendation occurred most frequently due to differences in the evaluation of the patients’ social situation (see Table 2).

**Table 2:** Reasons for deviations from the Level of Care (LOC) recommended based on the MATE in the intervention group

The staffs’ reason for deviation	Resulted in …		Total
	Higher LOC	Lower LOC	
Severity of addiction	10	4	14
Severity of psychiatric comorbidity	3	1	4
Severity of social disintegration	12	13	25
**Additional reasons reported by staff**			
Patient wants treatment at different LOC			1
Organizational problems on treatment unit			1
Lack of motivation			2
LOC does not fit the patients’ life circumstances			5
Lack of the patients’ capacity to undergo the recommended LOC			2

There was substantial agreement between the LOC recommended by the team and the result of the feedback session (κ=.80). In 11 patients, there was no agreement on a LOC. Another 19 patients had other preferences than the recommended LOC. Agreement between the LOC decision after the feedback session and the treatment patients actually received at follow-up was fair (κ=.25).

### Reactive and organizational factors affecting matching

From 165 patients responding to the follow-up assessment six months after the initial assessment interview, 125 patients reported any kind of aftercare since discharge from the qualified withdrawal treatment and were included in the following analyses. Single bivariate analyses revealed, that patients with a high psychiatric comorbidity (odds ratio OR=0.32; *p*=.014), with higher specific problem recognition (OR = 1.67; *p* = .034), and patients with a higher number of foregoing substance abuse treatment (OR = 1.21; *p* = .010) received more likely *undermatched* aftercare, whereas patients who were below the cut-off for social disintegration (OR=3.22; *p*=.031), patients with a lower number of foregoing substance abuse treatment (OR = 0.79; *p* = .036) as well as male patients (OR=3.61; *p*=.009) were more likely to receive overmatched aftercare (see Table 3). In the multifactorial regression, problem recognition and the number of foregoing substance abuse treatment remained statistically significant.

**Table 3:** Parameter estimates of the multinomial logistic regression analyses of matching as dependent variable investigating factors affecting matching beyond the intervention

	Bivariate analyses			Multifactorial analysis		
	***OR***	***95% CI***	***p***	***OR***	***95% CI***	***p***
**Undermatched**						
Problem recognition specific	***1.67***	***1.04 to 2.67***	***.034***	***2.35***	***1.06 to 5.21***	***.035***
Problem recognition general	1.54	0.90 to 2.61	.113	0.72	0.30 to 1.73	.469
Desire for help	1.34	0.75 to 2.44	.309	1.17	0.39 to 3.55	.780
Treatment readiness	0.93	0.55 to 1.58	.933	0.72	0.35 to 2.20	.775
Age	1.04	0.99 to 1.10	.082	1.06	0.99 to 1.12	.086
Informed^A^	0.70	0.17 to 2.89	.621	1.80	0.29 to 11.07	.527
SDM^A^	1.16	0.29 to 4.62	.835	2.26	0.37 to 13.48	.373
Severity of addiction	0.57	0.23 to 1.39	.213	1.34	0.39 to 4.54	.642
Severity of psychiatric comorbidity	***0.32***	***0.13 to 0.79***	***.014***	0.49	0.15 to 1.61	.240
Severity of social disintegration	0.64	0.27 to 1.52	.310	0.56	0.18 to 1.76	.319
Number of foregoing SAT	***1.21***	***1.06 to 1.38***	***.010***	***1.27***	***1.04 to 1.55***	***.018***
Male gender	0.83	0.36 to 1.95	.673	0.69	0.22 to 2.24	.531
Trial site 1^B^	0.77	0.26 to 2.32	.642	1.05	0.23 to 5.13	.951
Trial site 2^B^	1.23	0.35 to 4.31	.749	0.92	0.16 to 5.46	.931
Trial site 3^B^	1.23	0.35 to 4.31	.749	2.32	0.37 to 14.58	.368
**Overmatched**						
Problem recognition specific	1.55	0.96 to 2.45	.071	***2.35***	***1.08 to 5.11***	***.031***
Problem recognition general	1.13	0.70 to 1.84	.612	0.86	0.39 to 1.90	.702
Desire for help	0.96	0.54 to 1.71	.888	0.85	0.27 to 2.62	.771
Treatment readiness	1.05	0.61 to 1.80	.872	1.19	0.51 to 2.76	.684
Age	0.99	0.95 to1.04	.646	0.99	0.95 to 1.05	.891
Informed^A^	1.25	0.29 t0 5.35	.764	1.20	0.20 to 7.02	.841
SDM^A^	1.05	0.24 to 4.59	.946	1.11	0.18 to 6.78	.910
Severity of addiction	1.44	0.61 to 3.37	.404	0.97	0.32 to 2.94	.959
Severity of psychiatric comorbidity	2.12	0.87 to 5.16	.100	1.61	0.51 to 5.04	.415
Severity of Social Disintegration	***3.22***	***1.12 to 9.29***	***.031***	3.64	0.92 to 14.43	.066
Number of foregoing SAT	***0.79***	***0.64 to 0.99***	.***036***	***0.78***	***0.60 to 1.05***	***.054***
Male gender	***3.61***	***1.37 to 9.52***	***.009***	2.20	0.68 to 7.21	.190
Trial site 1^B^	0.33	0.11 to 1.02	.054	0.39	0.10 to 1.68	.210
Trial site 2^B^	0.88	0.27 to 2.89	.836	0.70	0.17 to 2.94	.629
Trial site 3^B^	0.53	0.15 to 1.93	.334	0.51	0.10 to 2.60	.418

*N* = 123, *OR* odds ratio, *95% CI* 95% confidence interval, *matched* was used as reference category; as independent variables age, gender, trial site, MFT scales, control preferences, and the MATE dimension scores history of substance use disorder treatments, severity of the addiction, severity of psychiatric comorbidity and severity of social disintegration were included in separate bivariate analyses as well as in one multifactorial analysis; ^A^Role preferences for either informed, shared or paternalistic decision making. Paternalistic decision making was used as reference category; ^C^Trial site 4 was used as reference category; Goodness of fit of the multifactorial model: *R*^2^_Cox&Snell_ = .468; *R*^2^_Nagelkerke_ = .527 .488

### Feasibility and acceptance of the intervention at trial site and potential for implementation

The expert workshop was attended by 11 participants from three of the four trial sites. Two participants from the fourth trial site could not attend personally and were interviewed by phone. The study procedure and intervention were regarded as feasible and acceptable by the participants. Recommendations derived by the MATE-interview were generally considered plausible and helpful. In Table 4, results of the expert workshop are summarized.

**Table 4:** Qualitative results of the expert workshop. Main categories including a description of subcategories are presented

***Acceptance of the MATE-Interview***	
• *Research assistants* felt comfortable in conducting the interview after an initial training period and perceived information they gathered by the MATE-assessment as useful • *Patients* seemed to appreciate the structure of the MATE-Interview including fully structured modules and modules with open questions; for some patients depending on physical and mental state, the MATE interview in combination with the CSSRI-EU seemed to be too demanding • *Staff of treatment unit (team)* appreciated feedback of the interview results	
**Acceptance of the study procedures**	
• *Research assistants* judged the study procedure, i.e. necessary assessments, material, and work flow, after initial training as being clear and acceptable. Training and ongoing supervision throughout the study was very helpful. Research assistants felt that the initial 2 days of training were too short to acquire all necessary skills for conducting the interviews and feedback sessions • *Patients* generally were interested in participation and seemed to benefit from study participation; • *The team* accepted the study procedures, while the perception of the PCMP implementation as study instead of daily routine was given in all participating treatment sites. Threats to the conduction were seen in high personnel fluctuation and also a high patient turnover	
**Cooperation of team and research assistants**	
• Cooperation of team and research assistants was seen as an essential agent of a successful implementation of the study. • *Good cooperation* lead to better exchange and integration of the information gathered within the study procedures and the course of treatment • *Communication barriers* occurred in namely in one of the participating sites, since parts of the team usually involved in treatment referral felt not sufficiently informed and feared to be “replaced” by PCPM	
**Plausibility of PCPM**	
• Recommendations calculated from the MATE-dimension scores in stage B of the PCPM were judged as reasonable, plausible and in many cases adequately matching the patients’ needs. • Communication of the recommendations within the team and to the patient (stage C) was judged as easy to understand and beneficial for the patient • Research assistants and team members perceived a high consistency between results of the PCPM and other clinical information regarding the patient • Threats to a reasonable use of PCPM during the study were especially seen in lacking coordination between the treatment as usual and the study procedures, especially regarding treatment referral	
**Implementation of PCPM in routine care**	
• *Potential benefits* in qualified withdrawal treatment: a higher transparency within the team and also between team and patient; a potential increase of quality in a setting with high personnel fluctuation was seen as major benefits when fully implemented in daily routine. • *Potential barriers* in qualified withdrawal treatment: the use of PCPM as realized in the study was seen as too time-consuming. Especially given a high patient turnover on these wards and a generally too little impact of treatment recommendations or arrangements for aftercare on the treatment patients actually receive afterwards, the effort of using PCPM was judged as too high by some of the participants • *Facilitators of implementation*: Several possibilities were discussed to reduce effort by maintaining the benefits of PCPM usage including a shortened version of the MATE for use in qualified withdrawal treatment and integration of the assessment results in already existing procedures as team meetings and regular visits • *Implementation in other settings*: drug counselling services and primary care were discussed as possible settings for an implementations of PCPM	

## Discussion

This process evaluation was conducted to gain a better understanding of the procedures and effect mechanisms of using PCPM in an inpatient qualified withdrawal program. Our objectives were to investigate consistency of the recommendations regarding aftercare throughout the different stages of the intervention, factors with a potential influence on the matching process for aftercare treatment and acceptance and feasibility of the use of PCPM.

The consistency between different stages of PCPM in the intervention group up to discharge from the qualified withdrawal treatment was moderate to substantial indicating plausibility and face validity of the recommendations calculated from the MATE, which was also supported by the results of the expert workshop. The most frequently reported deviation from the recommendation pertained to the severity of social disintegration, which could guide considerations regarding changes of the assessment and calculation of this MATE dimension score. In the feedback session itself, most deviations from the recommendation occurred due to different preferences of the patient. Since fostering an active role in decision-making was intended, this finding is in line with the aims of PCPM use. The workshop attendees did also emphasize the potential of the PCPM to foster transparency in team communication as well as shared decision making.

Compared to LOC1 and LOC4, we found the highest proportion of recommendations to LOC2 and LOC3 by the MATE. This tendency even increased during the process of PCPM in the intervention group, but was also observed in both groups at follow-up. With regard to the recommendations by the MATE-interview, we found the same pattern in one of our pilot studies [21]. This may reflect a higher problem severity of patients following an inpatient qualified withdrawal treatment compared with the Dutch approach, where the allocation is applied at the front door of any substance abuse treatment. The increase of this tendency in the course of PCPM use and treatment referral may stem from lacking treatment opportunities in LOC1 or LOC2. On the long term, this observation may guide decisions regarding changes in the range of treatments that can be offered. In the use of PCPM for an individual patient, it may lead to an adjustment of a LOC-recommendation. Especially because the proportion of patients in LOC1 and LOC4 is low, we think PCPM is useful to detect those patients.

Despite the consistency of recommendations in the intervention group up to patients’ discharge, there was no difference between the intervention group and control group regarding the proportion of matched patients. The use of PCPM apparently failed to affect the actual referral to aftercare. We concluded that there are too many other factors having an effect on treatment referral [23]. In this study, we found the number of foregoing substance abuse treatments as a predictor for being either under- or overmatched. Our findings regarding factors influencing whether patients received matched or mismatched aftercare could be associated with barriers to access substance abuse treatment or lacking suitable treatment services for subgroups like patients with comorbid disorders [32] or patients with a high number of previous treatments, but should be interpreted with caution due to the lack of statistical power.

The results of our expert workshop imply that we did not succeed in implementing PCPM at the four trial sites into daily routine. Due to the study design using randomizing on patient level, treatment units could not accommodate the procedure to all of their patients. Furthermore, for organizational reasons, all assessments and the intervention itself were conducted by a research assistant who was not part of the multidisciplinary team. On the other hand, the consistency of the different stages of the PCPM was still moderate to substantial – therefore we assume, that the unclear referral process after treatment is the major challenge in order to reach a higher percentage of matched treatments.

Regarding a successful implementation of the PCPM in routine care, the duration of the assessment interview as well as the whole procedure seems crucial. From preparatory studies we know that the duration of the MATE interview in a comparable setting can vary between 30 and 90 minutes with a mean of 45 minutes [21]. In our expert workshop, this was seen as one possible barrier for the implementation of the PCPM into routine care. However, in the meantime, a self-report version of the MATE has been developed showing acceptable concurrent validity [33]. A German version has been translated and is currently being validated for its use to facilitate referral decisions [34]. This version could facilitate an implementation into routine care in the future.

In the interpretation of our results, there are limitations to consider. Since we included only four trial sites, regional variation in the structure of substance abuse treatment could not systematically be integrated in our process evaluation, which would have been important especially for a closer investigation of factors having an influence on referral to aftercare. Furthermore, we did not include a measure of patients’ acceptance of the procedure and outcomes of the PCPM. In order to emphasize the patient-centeredness of this approach, this should be addressed in future studies. Choosing a trial design with randomization on patient level led to problems in the implementation of PCPM, where other studies using a naturalistic design with all patients being included in the new approach succeeded in the implementation of the respective matching approach [7, 15]. However, an opportunity to combine a rigorous methodological approach with the advantage of a full implementation at each participating treatment site may be the stepped wedge design [35], where randomization takes place on the level of treatment sites.

## Conclusion

Given the partial support of our underlying matching hypotheses [23], the promising results regarding face validity, plausibility, acceptance, and its potential to improve transparency and patient-centeredness we believe that the use of PCPM can improve the quality of referral decision-making from qualified withdrawal treatment to aftercare. Whether the use of PCPM can have an effect of health outcomes and costs, cannot be decided from our results and should be addressed in further studies. A combination with integrated care models or other means to make referral decisions more binding seems to be necessary to assure a higher proportion of matched patients.

## Data Availability

The datasets used and/or analysed during the current study are available from the corresponding author on reasonable request.

## Abbreviations

PCPM: Patient-centered matching guidelines
LOC: Level of care
MATE: Measurements in the Addictions for Triage and Evaluation
CSSRI-EU: Client Sociodemographic and Service Receipt Inventory
MFT: Motivation for Treatment Scale
OR: Odds ratio
